# Complete genome sequence of *Bradyrhizobium ottawaense* strain MIAE 01942 isolated from soybean nodules grown in antibiotic-amended soil

**DOI:** 10.1128/mra.00004-24

**Published:** 2024-03-19

**Authors:** Andrew Scott, Edward Topp, Cécile Revellin, Alain Hartmann, Michael Fruci

**Affiliations:** 1 Agriculture and Agri-Food Canada, London Research and Development Centre, London, Ontario, Canada; 2 Agroécologie Research Unit, INRAE, Université de Bourgogne, Dijon, France; 3 Department of Biology, University of Western Ontario, London, Ontario, Canada; 4 Department of Microbiology and Immunology, University of Western Ontario, London, Ontario, Canada; University of Maryland School of Medicine, Baltimore, Maryland, USA

**Keywords:** nodulation, legume symbionts, nitrogen-fixation, nitrous oxide reduction, *Bradyrhizobium*

## Abstract

*Bradyrhizobium ottawaense* MIAE 01942 is a symbiotic nitrogen-fixing bacterium isolated from the root nodules of soybeans grown in agricultural soils amended with veterinary antibiotics. The genome consists of a single 8.45 Mb circular chromosome that harbors genes involved in nitrogen fixation, denitrification, and antibiotic and metal resistance.

## ANNOUNCEMENT

Application of symbiotic, nitrogen-fixing, and nitrous oxide (N_2_O)-reducing bradyrhizobial inoculants in soybean production is a promising approach to minimizing the environmental impacts of inorganic nitrogen fertilizer use and N_2_O emissions (1, 2). Previously, Revellin et al. isolated a symbiotic bacterium from nitrogen-fixing soybean root nodules obtained from experimental microplots in London, Canada, which were treated with antibiotics used in swine production, including tylosin, chlortetracycline, and sulfamethazine (3). Phylogenetic analysis based on multilocus sequence typing, 16S rRNA gene and intergenic spacer sequencing, identified the bacterium as a strain of *Bradyrhizobium ottawaense* (designated strain MIAE 01942) (3). Importantly, this species has great potential for N_2_O mitigation in agricultural soils, owing to the high expression levels of an N_2_O reductase (encoded by *nosZ*) (4). Here, we report the complete genome sequence of *B. ottawaense* strain MIAE 01942.

*B. ottawaense* MIAE 01942 cells were grown at 28°C in yeast extract mannitol broth. Genomic DNA was extracted using the Biosearch Technologies MasterPure Total DNA and RNA Purification Kit. DNA quantity and quality were determined using a NanoDrop spectrophotometer and a Qubit 3.0 fluorometer. A genomic library was prepared from unsheared DNA using the Oxford Nanopore Technologies Native Barcoding Ligation Sequencing Kit v.14, SQK-NBD114-24, and the supplied Long Fragment Buffer to size-select fragments >3 Kb. Sequencing was performed on a MinION Mk1B using a R10.4.1 flow cell. Default parameters were used for all software, unless otherwise stated. Reads were basecalled using high accuracy basecalling (HAC) mode (*Q*-value of 9), and demultiplexed with MinKNOW v.23.04.3. Reads were further filtered and trimmed using Filtlong v.0.2.1 (https://github.com/rrwick/Filtlong) with additional parameters (--min_length 1000 --keep_percent 90), producing 248,283 reads (3.009 Gbp, N_50_ = 21,806 bp). *De novo* assembly using Flye v.2.9.2 (5) specifying Nanopore reads followed by polishing with Medaka v.1.8.0 (https://github.com/nanoporetech/medaka) yielded a circular contig with 352 times coverage.

**Fig 1:**
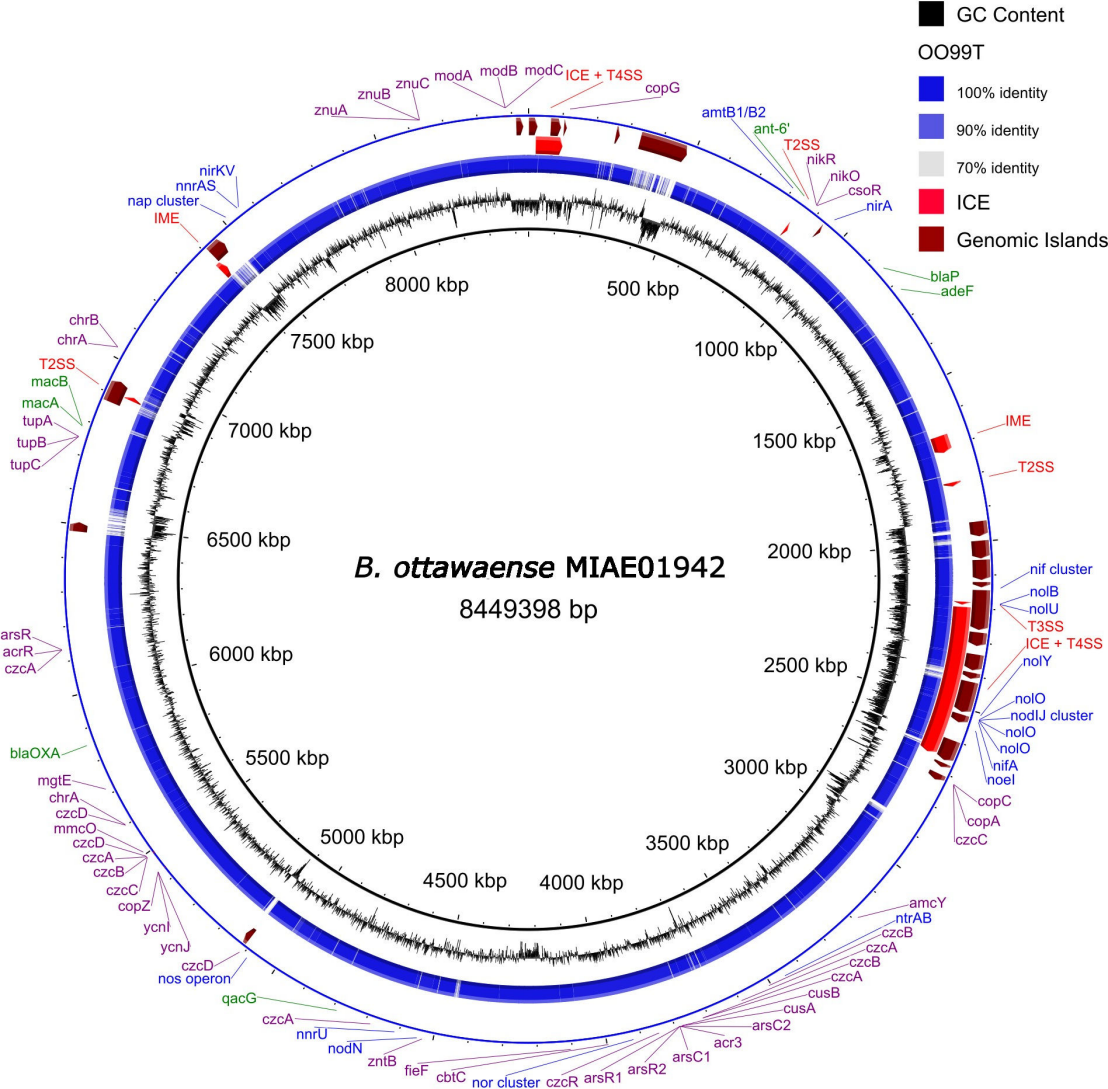
Circular map of the complete genome of *B. ottawaense* MIAE 01942. The innermost ring (black) shows the GC content of the *B. ottawaense* MIAE 01942 genome. The blue ring illustrates the percent identity of the selected *B. ottawaense* OO99^T^ genome with the *B. ottawaense* MIAE 01942 genome calculated using blastn (*E*-value = 10, lower identity cutoff of 70% and upper identity cutoff of 90%). The red ring shows integrative conjugative elements (ICE) and integrated and mobilizable elements (IME). The maroon ring illustrates genomic islands. The outermost ring displays selected genes color-coded as follows: nitrogen fixation, nodulation, and denitrification genes in blue, antibiotic resistance genes in green, metal resistance genes in purple, and secretion systems in red. The circular map was generated using BLAST Ring Image Generator software v.0.95 (7).

The complete genome of *B. ottawaense* MIAE 01942 comprises a single circular chromosome of 8,449,398 bp (G + C content of 63.83%) (Fig. 1). A total of 7,858 genes, 7,582 protein coding sequences, 51 tRNAs, and 3 rRNA genes were found with the National Center for Biotechnology Information (NCBI) Prokaryotic Genome Annotations Pipeline (PGAP) v.6.6. Nodulation, nitrogen fixation, denitrification (including *nosZ*), metal resistance, and type I to IV secretion system genes were found. Seven antibiotic resistance genes were detected using RGI-*bwt* v.3.1.4 and ABRicate (https://github.com/tseemann/abricate) with the CARD database v.3.2.4 (6).

Twenty-four putative genomic islands were identified using IslandViewer 4 and the IslandPath-DIMoB prediction method (8). The *nod*, *nif*, and type III secretion system genes co-localized to form a symbiosis island. Two putative integrative conjugative elements, both of which contain type IV secretion system gene clusters, and two integrative mobile elements were detected using ICEBerg v.3.0 (9).

This work provides genetic insights into the species, *B. ottawaense*, which has the potential for reducing both inorganic fertilizer use and N_2_O emissions in soybean production.

## Data Availability

The complete genome sequence of *B. ottawaense* MIAE 01942 was deposited in GenBank under the accession number CP140754. Raw sequences have been deposited in the NCBI Sequence Read Archive under the BioProject and SRA accession numbers PRJNA1051999 and SRR27197669, respectively.
